# Different Types of Cell Death in Diabetic Neuropathy: A Focus on Mechanisms and Therapeutic Strategies

**DOI:** 10.3390/ijms25158126

**Published:** 2024-07-25

**Authors:** Shang Ye, Zilin Cheng, Dongye Zhuo, Shuangmei Liu

**Affiliations:** 1 Department of Clinical Medicine, School of Queen Mary, Jiangxi Medical College, Nanchang University, Nanchang 330031, China; yeshang2001@163.com (S.Y.); czl1303561190@163.com (Z.C.); conor.upup@gmail.com (D.Z.); 2 Department of Physiology, School of Basic Medicine, Jiangxi Medical College, Nanchang University, Nanchang 330031, China

**Keywords:** diabetic neuropathy, cell death, apoptosis, autophagy, pyroptosis, ferroptosis

## Abstract

Diabetic neuropathy (DN) is a common complication of diabetes, affecting over 50% of patients, leading to significant pain and a burden. Currently, there are no effective treatments available. Cell death is considered a key factor in promoting the progression of DN. This article reviews how cell death is initiated in DN, emphasizing the critical roles of oxidative stress, mitochondrial dysfunction, inflammation, endoplasmic reticulum stress, and autophagy. Additionally, we thoroughly summarize the mechanisms of cell death that may be involved in the pathogenesis of DN, including apoptosis, autophagy, pyroptosis, and ferroptosis, among others, as well as potential therapeutic targets offered by these death mechanisms. This provides potential pathways for the prevention and treatment of diabetic neuropathy in the future.

## 1. Introduction

Diabetic neuropathy (DN) is a common complication in diabetic patients, as hyperglycemia can cause irreversible nerve damage [1]. Over 50% of diabetic patients will develop varying degrees of neuropathy [2]. According to the position statement of the American Diabetes Association, DN can be categorized into diffuse neuropathy, mononeuropathy, and radiculopathy or polyradiculopathy. Diffuse neuropathy is further divided into distal symmetric polyneuropathy (DSPN) and autonomic neuropathy [3]. DSPN accounts for about 75% of DN and is the typical form of diabetic peripheral neuropathy (DPN) [4]. DPN can impact both sensory and motor nerves, leading to diverse clinical manifestations. Some patients may be asymptomatic, whereas others may exhibit one or more symptoms, such as paresthesia, numbness, and neuropathic pain. These symptoms can vary in intensity from mild to persistent and cause substantial distress [5]. Diabetic autonomic neuropathy can impact nearly all autonomic nerves, with symptoms varying according to the affected organ or system. This condition may result in arrhythmia, asymptomatic ischemia, myocardial infarction, gastroparesis, constipation, diabetic bladder dysfunction, sexual dysfunction, and sudomotor dysfunction, significantly increasing patient suffering, their burden, and the risk of mortality. Even worse, diabetes also damages the central nervous system, leading to cognitive decline, dementia, and diabetic encephalopathy [6]. Despite the fact that DN seriously affects the quality of life of patients, there is no efficient intervention available.

Cell death, from a physiological perspective, serves as a crucial mechanism for maintaining the homeostasis of tissue and organ functions. However, aberrant cell death can contribute to the development and progression of a wide range of diseases. Briefly, based on morphological criteria, cell death can be classified into four distinct types. Type I, known as apoptosis, is characterized by cell shrinkage, DNA fragmentation, chromatin condensation, membrane blebbing, and the formation of an apoptosome. Type II, referred to as autophagy, is marked by extensive vacuolization of the cytoplasm, leading to the formation of autophagosomes, followed by phagocytosis and lysosomal degradation. In these two types of cell death, the plasma membrane remains intact. Meanwhile, type III cell death, or necrosis, is morphologically characterized by organelle swelling and rupture of the plasma membrane, resulting in the release of cellular contents without evident phagocytosis or lysosomal degradation. Type IV, known as entosis, involves one living cell engulfing and killing another cell of the same type [7]. In recent years, an increasing number of studies have focused on the role of various cell death mechanisms in DN, unveiling the impact of multiple forms of cell death in DN and exploring avenues for alleviating DN by targeting these death pathways. The intricate relationship between cell death pathways and DN offers a promising approach for therapeutic intervention.

Here, we comprehensively review how cell death is triggered in diabetic neuropathy and the methods to alleviate cell death by targeting these pathways, with the aim of developing innovative therapeutic strategies for DN.

## 2. Mechanisms of Cell Death Involved in DN

**Figure 1:**
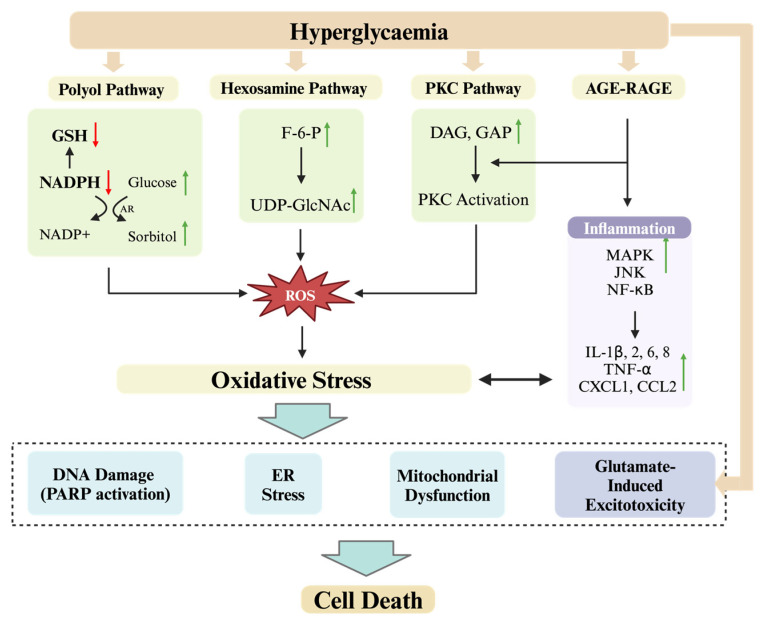
Pathways leading to cell death in DN. Hyperglycemia activates several metabolic pathways: the polyol pathway, hexosamine pathway, PKC pathway, and AGE-RAGE pathway. In the polyol pathway, hyperglycemia increases glucose flux, converting glucose to sorbitol via AR, which consumes NADPH and reduces GSH regeneration, leading to oxidative stress. The hexosamine pathway increases UDP-GlcNAc, contributing to advanced glycation end-products (AGEs) and enhancing oxidative stress. The PKC pathway is activated by elevated DAG and GAP, promoting inflammation and oxidative stress. The AGE-RAGE pathway activates signaling pathways (MAPK, JNK, NF-κB), increasing inflammatory cytokines (IL-1β, IL-2, IL-6, IL-8, TNF-α, CXCL1, CCL2). Hyperglycemia also directly leads to glutamate-induced excitotoxicity, contributing to neuronal damage. These pathways converge to produce ROS, causing oxidative stress, DNA damage (via PARP activation), ER stress, mitochondrial dysfunction, and glutamate-induced excitotoxicity, ultimately leading to neuronal cell death. Red arrows indicate a decrease in certain molecules or pathways, green arrows indicate an increase in certain molecules or pathways. GSH, glutathione; NADPH, nicotinamide adenine dinucleotide phosphate (reduced form); AR, aldose reductase; F-6-P, fructose-6-phosphate; UDP-GlcNAc, UDP-N-acetylglucosamine; DAG, diacylglycerol; GAP, glyceraldehyde-3-phosphate; PKC, protein kinase C; AGE, advanced glycation end-product; RAGE, receptor for advanced glycation end-products; MAPK, mitogen-activated protein kinase; JNK, c-Jun N-terminal kinase; NF-κB, nuclear factor kappa-light-chain-enhancer of activated B cells; IL, interleukin; TNF-α, tumor necrosis factor-alpha; CXCL1, C-X-C motif chemokine ligand 1; CCL2, C-C motif chemokine ligand 2; ROS, reactive oxygen species; PARP, poly (ADP-ribose) polymerase.

Diabetes is a metabolic disorder mainly characterized by an imbalance in glucose homeostasis, leading to hyperglycemia. This glucose imbalance is closely related to other metabolic disorders. For example, diabetes also impacts lipid metabolism, leading to dyslipidemia. These metabolic imbalances affect and disrupt many pathways. Over the past decades, researchers have focused on the molecular pathways associated with diabetes-induced neuropathy. Previous research has identified several key mechanisms contributing to cell death in DN, including oxidative stress, mitochondrial dysfunction, inflammation, endoplasmic reticulum (ER) stress, and glutamate-induced excitotoxicity (Figure 1).

### 2.1. Oxidative Stress

Oxidative stress arises when the production of reactive oxygen species (ROS) exceeds antioxidant defense mechanisms, potentially causing damage. This imbalance is pivotal in the development of various diseases, including cancer and neurodegenerative diseases [8].

Under normal conditions, glucose is progressively phosphorylated and metabolized through the glycolytic process, generating products such as glucose-6-phosphate, fructose-6-phosphate, glyceraldehyde-3-phosphate (GAP), and pyruvate [9]. However, in diabetes, excessive blood glucose levels lead to an increase in the production of these intermediates, thereby activating various pathways that result in the overproduction of ROS. Hyperglycemic conditions result in an excessive influx of glucose into the polyol pathway, where it is converted into sorbitol by aldose reductase, accumulating within nerve fibers, leading to cellular osmotic imbalance. During this reduction process, there is a significant consumption of NADPH (nicotinamide adenine dinucleotide phosphate), which plays a crucial role in maintaining the cellular redox cycle. NADPH is essential for the regeneration of the endogenous free radical scavenging antioxidant GSH (glutathione). The substantial depletion of NADPH results in a reduction in GSH, thereby elevating intracellular ROS levels [10]. Concurrently, activation of the polyol pathway enhances fructose production, subsequently increasing levels of GAP and dihydroxyacetone phosphate. This elevation in GAP and dihydroxyacetone phosphate stimulates the protein kinase C pathway, further amplifying ROS generation [11]. Additionally, during glycolysis, the surplus production of fructose-6-phosphate is channeled through the hexosamine pathway, culminating in the formation of UDP-N-acetylglucosamine. The accumulation of excessive UDP-N-acetylglucosamine leads to hyperactivity of O-glucosamine-N-acetyl transferase, resulting in toxicity and pro-oxidative effects [12]. Moreover, the intermediate GAP in glycolysis can be converted to diacylglycerol, which activates the protein kinase C pathway in neurons [13]. Its activation leads to the production of ROS, which exacerbates the level of oxidative stress in neuronal cells [14]. Furthermore, exposure of proteins and lipids to elevated glucose levels results in their glycation, forming highly reactive compounds known as advanced glycation end-products (AGEs) [15]. Accumulation of AGEs in various tissues and organs is associated with the development of DN. The pathophysiological processes of AGEs are divided into AGE receptor-dependent and AGE receptor-independent mechanisms [16]. In the AGE receptor-dependent pathway, the interaction between AGEs and AGE receptors upregulates the expression of pro-inflammatory cytokines through various pathways including the AGE–AGE receptor–NF-κB axis, c-Jun-N-terminal kinase (JNK), and the p38 mitogen-activated protein kinase (MAPK) pathway [17]. This interaction leading to an increase in intracellular inflammatory responses and ROS [18]. In the AGE receptor-independent pathway, AGEs can directly lead to increased ROS levels. Furthermore, AGEs can upregulate the level of CCAAT-enhancer-binding protein homologous protein (CHOP) to induce ER stress, which can lead to cell death [16]. Excessive levels of ROS can inflict damage upon biomolecules such as cellular DNA, leading to the activation of poly (ADP-ribose) polymerase 1 (PARP-1) [19]. Uncontrolled activation of PARP can lead to various cell death mechanisms, including apoptosis, pyroptosis, and parthanatos, among others [20]. Oxidative stress can also induce cell death by triggering mitochondrial dysfunction, ER stress, and changes in autophagy levels, among other mechanisms.

### 2.2. Mitochondrial Dysfunction

Mitochondrial dysfunction refers to a range of conditions characterized by abnormalities in mitochondrial function. This can include alterations in the expression levels of mitochondrial markers at the mRNA and protein levels, changes in the activity of the mitochondrial electron transport chain (ETC), fluctuations in mitochondrial membrane potential (MMP), variations in the enzymatic activities of components involved in mitochondrial oxidation processes, and disruptions in mitochondrial trafficking [21]. Under physiological conditions, glucose is metabolized to produce NADH, which donates electrons to the ETC, facilitating ATP production. However, in diabetes, in addition to the various ROS-producing pathways activated during glycolysis, hyperglycemia leads to increased production of pyruvate, which leads to an increased flux through the ETC, resulting in excessive production of ROS [18]. Concurrently, this condition triggers a hyperpolarization of the MMP, which implies an increase in the mitochondrial energy state. However, this state is quickly followed by a depolarization of the MMP, a process that is closely associated with an increase in the ADP:ATP ratio and a significant decrease in ATP levels, reflecting a compromise in the mitochondrial ability to generate energy [22]. Excessive ROS production can damage mitochondrial components, including lipids, proteins, and mitochondrial DNA, leading to mitochondrial dysfunction. This dysfunction not only undermines ATP production but also amplifies ROS generation, engendering a detrimental loop of mitochondrial impairment and oxidative stress [23]. Subsequently, these mitochondrial perturbations lead to the release of cytochrome c from the mitochondrial intermembrane space, activating caspases and leading to neuron death [24].

### 2.3. Inflammation 

Inflammation also plays an important role in causing cell death in DN. In diabetes, oxidative stress can activate multiple downstream signaling pathways, such as MAPK, JNK, and NF-κB [25]. In addition, interactions between AGEs and AGE receptors can also activate NF-κB. The activation of these pathways triggers a cascade of events leading to the production of pro-inflammatory cytokines and chemokines, including interleukin-1 beta (IL-1β), IL-2, IL-6, IL-8, tumor necrosis factor-alpha (TNF-α), chemokine (C-X-C motif) ligand 1 (CXCL1), and chemokine (C-C motif) ligand 2 (CCL2) [26]. These inflammatory mediators not only enhance existing inflammatory and immune responses but also increase cellular oxidative stress, thereby exacerbating neuronal damage in DN [27]. The intricate interplay between inflammation and oxidative stress promotes various modes of cell death, thus highlighting the complex pathophysiology of DN.

### 2.4. ER Stress

**Figure 2:**
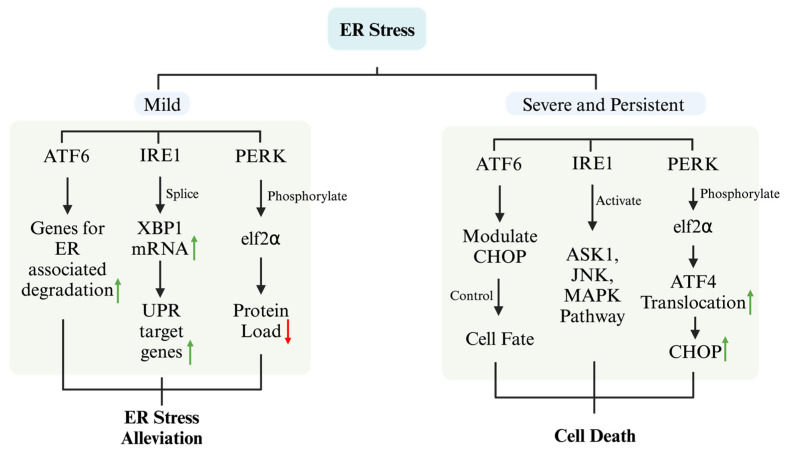
Mechanisms of ER stress in the cellular response. In mild ER stress, three primary signaling pathways are activated: the ATF6 pathway, which upregulates genes involved in ER-associated degradation to reduce the load of misfolded proteins; the IRE1 pathway, which splices XBP1 mRNA, leading to the production of UPR target genes that enhance protein folding capacity; and the PERK pathway, which phosphorylates eIF2α, reducing protein synthesis to decrease the protein load entering the ER. Under severe and persistent ER stress, these pathways lead to different outcomes: the ATF6 pathway modulates the expression of CHOP, influencing cell fate; the IRE1 pathway activates ASK1, JNK, and the MAPK pathway, leading to apoptosis; and the PERK pathway’s phosphorylation of eIF2α promotes the translocation of ATF4, upregulating CHOP and contributing to apoptotic signaling. This dual response mechanism shows how cells attempt to restore homeostasis under mild ER stress but may trigger apoptotic pathways if the stress is severe and prolonged, ultimately determining the cell fate. Red arrows indicate a decrease in certain molecules or pathways, green arrows indicate an increase in certain molecules or pathways. ATF6, activating transcription factor 6; IRE1, inositol-requiring enzyme 1; XBP1, X-box binding protein 1; PERK, protein kinase RNA-like ER kinase; eIF2α, eukaryotic initiation factor 2-alpha; UPR, unfolded protein response; ASK1, apoptosis signal-regulating kinase 1; JNK, c-Jun N-terminal kinase; MAPK, mitogen-activated protein kinase; CHOP, C/EBP homologous protein; ATF4, activating transcription factor 4.

The ER is recognized as one of the largest organelles within eukaryotic cells, playing a pivotal role in the synthesis, folding, and structural formation of proteins. Moreover, it is now widely recognized that ER is not only involved in protein processing but also plays an important role in a range of cellular activities, including lipid synthesis, linkage of cytoskeletal structures, transport of molecules, signaling, and regulation of Ca^2+^ homeostasis [28]. During protein synthesis, when proteins misfold or fail to fold into a normal structure and build up in the ER beyond the capacity of the ER, ER stress and the unfolded protein response (UPR) are activated [29]. The UPR is a complex signaling network that aims to restore normal function and homeostasis by halting protein translation, degrading misfolded proteins, and increasing the production of molecular chaperones that contribute to protein folding [30]. The UPR is predominantly regulated by transmembrane proteins: activating transcription factor 6 (ATF6), protein kinase RNA-activated (PKR)-like endoplasmic reticulum kinase (PERK), and inositol-requiring enzyme 1 (IRE1) (Figure 2) [31]. Under mild ER stress, these three transmembrane proteins can activate downstream transcription factors that are translocated into the nucleus, thereby regulating the protein folding capacity of the ER and thus facilitating the recovery from stress. The activation of ATF6 upregulates genes associated with ER-associated degradation, reducing ER stress. IRE1 splices X-box binding protein 1 (XBP1) mRNA can upregulate UPR target genes to mitigate ER stress. However, under severe and persistent ER stress, these three transmembrane proteins can activate cell death pathways. ATF6 contributes to the determination of the cell fate by modulating CHOP [32]. IRE1 exhibits endoribonuclease activity and also targets additional mRNAs through the mechanism of regulated IRE1-dependent decay, an event that may facilitate cell death. Moreover, IRE1 can activate apoptosis signal-regulating kinase 1, thereby activating downstream JNK and MAPK pathways to promote apoptosis [33]. The activation of PERK leads to the phosphorylation of the eukaryotic translation initiation factor-2 alpha (eIF2α), which serves to reduce the protein load entering the ER, thereby alleviating stress [34]. However, the phosphorylation of eIF2α also selectively enhances the translation of ATF4 and then promotes the expression of the pro-apoptotic molecule CHOP, thereby facilitating cell death [35]. In a cellular model of DN, glucose and palmitate synergistically increase the levels of XBP1 and CHOP in RSC96 cells and promote the phosphorylation of eIF2α, thereby inducing apoptosis [36]. Furthermore, an increased ratio of CHOP/oxygen-regulated protein 150 (ORP150) facilitates cell apoptosis in DN [37], indicating that changes in CHOP levels induced by ER stress play a significant role in the initiation of cell death. 

### 2.5. Glutamate-Induced Excitotoxicity

Glutamate is a crucial excitatory neurotransmitter in the central nervous system, and its activity in the synaptic cleft is balanced through receptor inactivation and glutamate reuptake. When this balance is disrupted, excessive glutamate can lead to excitotoxicity [38]. Glutamate receptors are classified into metabotropic glutamate receptors and ionotropic glutamate receptors. The neurotransmitter glutamate primarily acts through ionotropic glutamate receptors, which include α-amino-3-hydroxy-5-methyl-4-isoxazolepropionic acid (AMPA) receptors, kainate receptors, and N-methyl-D-aspartate (NMDA) receptors. Notably, overactivation of NMDA receptors can cause excitotoxicity, leading to intracellular Ca^2+^ overload and subsequent cell death [39]. This mechanism has been identified in conditions such as ischemia, stroke, and Alzheimer’s disease [40,41]. In DN, metabolic disturbances caused by hyperglycemia can lead to changes in neurotransmitter levels. A study found that in the cerebral cortex of streptozotocin (STZ)-induced diabetic rats, there was an upregulation of AMPA and NMDA receptor density, as well as increased glutamate levels, which triggered excitotoxic neuronal death [42]. This suggests that glutamate-induced excitotoxicity may also be one of the mechanisms leading to cell death in DN.

## 3. Types of Cell Death and Potential Targets in DN

### 3.1. Apoptosis in DN

Apoptosis, a programmed cell death, is a fundamental biological process that plays a crucial role in the development, maintenance, and health of multicellular organisms. Apoptosis can be induced via two pathways: the intrinsic (also known as the mitochondrial) pathway, and the extrinsic (death receptor) pathway. The intrinsic pathway is primarily governed by the mitochondria and is triggered by internal stimuli such as DNA damage, oxidative stress, and other cellular stressors. This pathway is characterized by mitochondrial outer membrane permeabilization, which is predominantly regulated by members of the B-cell lymphoma 2 (BCL-2) protein family. This family includes pro-apoptotic proteins (such as Bax and Bak), anti-apoptotic proteins (such as Bcl-2 and Bcl-xL), and pro-apoptotic BCL-2 homology 3 (BH3)-only proteins (such as Bad, Bid, and p53 upregulated modulator of apoptosis) [43]. The pivotal event in this pathway is the translocation of cytochrome c from the mitochondrial intermembrane space into the cytosol. Once released, cytochrome c associates with apoptotic protease-activating factor 1 and procaspase-9 to assemble the apoptosome. This complex formation initiates the activation of caspase-9, which in turn activates downstream effector caspase-3/7, thereby executing apoptosis [44]. The extrinsic pathway is initiated by the binding of extracellular death ligands to their corresponding death receptors on the cell surface, such as Fas ligand to the Fas receptor or TNF-α to the TNF receptor. This interaction leads to the activation of procaspase-8 into caspase-8, which subsequently activates caspase-3/7, leading to cell death. In addition, the extrinsic apoptosis pathway can trigger the intrinsic pathway through caspase-8-mediated cleavage of Bid, a BH3-only protein, thus amplifying the apoptotic response by linking these two pathways [45]. 

In DN, apoptosis can occur in both neurons and glial cells. For example, apoptosis of Schwann cells (SCs) and dorsal root ganglia (DRG) neurons was identified decades ago in modeling studies [46]. SCs are specialized glial cells localized to the peripheral nervous system. Their primary functions include the formation of myelin sheaths, which insulate axons, thereby optimizing the transmission of neural signals. Furthermore, SCs secrete an array of neurotrophic factors and essential nutrients, pivotal for axonal regeneration and repair following nerve injury [47]. Apoptosis of SCs causes demyelination, neuronal damage, axonal degeneration, nerve regeneration damage, and chronic neuropathic pain [48]. In DN, experimental studies have shown that high glucose levels promote apoptosis in RSC96 cells, an in vitro model of SC [49]. Histological analysis of sciatic nerve biopsies from diabetic rodents, cats, and patients has revealed a decrease in the density of myelinated nerve fibers, which may be related to the apoptosis of SCs and neurons [50,51]. DRG are the convergence points for the cell bodies of primary sensory neurons, playing a critical role in the transmission and modulation of somatic sensations, as well as in the reception and conduction of nociceptive stimuli. Apoptosis of small nerve fibers in the DRG can lead to neuropathic pain, while apoptosis of large nerve fibers can result in numbness and hyperalgesia. 

In addition to occurring in the peripheral nervous system, apoptosis can also occur in various types of cells in the nervous system, including hippocampal neurons and enteric glial cells, leading to corresponding symptoms such as cognitive impairment and gastrointestinal dysfunctions [52,53]. Persistent hyperglycemia and metabolic disturbances in diabetes can induce significant apoptotic activity in the dorsal horn neurons, leading to central sensitization and the development of chronic neuropathic pain. Studies using rat models have shown significant changes in the expression of apoptosis-related genes in the dorsal horn [54]. Another study further elucidated the relationship between apoptosis in the lumbar dorsal horn and chronic neuropathic pain by demonstrating that a metabotropic glutamate receptor 5 antagonist reduces Bax expression and neuronal apoptosis, thereby alleviating hyperalgesia [55]. In addition, STZ-induced apoptosis of spinal dorsal horn neurons in diabetic rats was increased, manifested by the activation of caspase-3 and an increase in the Bax/Bcl2 ratio, indicating that diabetes can lead to apoptosis of spinal cord neurons. Satureja khuzestanica attenuates apoptosis in the spinal cord of diabetic rats, indicating that Satureja has the therapeutic potential to attenuate diabetes neuropathy [56]. Losartan, as an angiotensin II Type 1 receptor antagonist, can reduce the apoptosis of spinal cord neurons caused by diabetes through the activation of the nuclear factor erythroid 2-related factor 2/heme oxygenase 1 system (Nrf2/HO-1) [57].

Currently, strategies to alleviate cell apoptosis in DN primarily focus on addressing the mechanisms leading to apoptosis, such as antioxidation to directly reduce ROS, inhibiting pathways that produce ROS [58], reducing ER stress, and alleviating mitochondrial dysfunction and anti-inflammation.

For reducing ROS, alpha-lipoic acid (ALA), as one of the most representative antioxidants directly targeting ROS, has been extensively studied for its potential therapeutic effects in DN. Several experiments in rats have demonstrated that ALA can reduce neuronal apoptosis in DN by mitigating oxidative stress, as well as decrease demyelination [59,60,61]. Previous studies have also considered the clinical application of ALA to be effective [62]. However, a recent meta-analysis suggested that the efficacy of alpha-lipoic acid (ALA) in alleviating various typical symptoms of DN, including neuropathic pain and numbness, in patients with type 1 and type 2 diabetes is minimal [63]. This discrepancy suggests that the clinical application and efficacy of ALA require further evaluation. Research studies indicate that IL-10 and vitamin D can suppress the activation of the NF-κB pathway by AGEs to reduce the accumulation of ROS within SCs, thereby diminishing the incidence of apoptosis [64,65]. Polydatin can inhibit the expression of the AGE receptor and activate the Nrf2 pathway to reduce the toxicity of AGEs, thereby producing a similar effect in alleviating SCs’ apoptosis [66]. Direct supplementation of GSH in the body is also a potential therapeutic approach. Clinical studies have shown that long-term oral administration of GSH can help elderly patients with type 2 diabetes achieve better blood sugar control [67]. Stable blood sugar control is beneficial in reducing the incidence of DN.

In regard to alleviating mitochondrial dysfunction, the sirtuin (SIRT) family, a class of NAD+-dependent histone deacetylases, has been shown to play a key role in a variety of cellular processes, including inflammation, metabolism, and oxidative stress. SIRT3-5 are localized within the mitochondria and play a significant role in mediating cell apoptosis and mitochondrial dysfunction [68]. Current research focused on inhibiting apoptosis in DN by targeting mitochondrial dysfunction predominantly concentrates on pathways associated with SIRT. A recent study discovered that dexmedetomidine treatment alleviates mitochondrial dysfunction and reduces SCs’ apoptosis by downregulating microRNA-34a, thereby upregulating SIRT2 and inhibiting the expression of S1PR1 [69]. Another study identified that formononetin activates SIRT3, which in turn upregulates superoxide dismutase 2 and peroxisome proliferator-activated receptor gamma coactivator 1-alpha, inhibiting mitochondrial dysfunction and reducing SCs’ apoptosis [70]. In addition to targeting SIRT, alleviation of mitochondrial dysfunction can also be achieved by reducing mitochondrial overload. Pyruvate dehydrogenase kinase inhibits the conversion of pyruvate to acetyl-CoA, thereby alleviating mitochondrial overload. Studies indicate that in the hippocampal neurons of STZ-induced diabetic mice, downregulation of pyruvate dehydrogenase kinase and promoting the expression of pyruvate can provide neuroprotective effects [71].

The idea of reducing ER stress is mainly to inhibit UPR-related pathways as well as downstream signaling. In regard to targeting the PERK pathway, research has demonstrated that using the PERK inhibitor GSK2606414 can counteract hyperglycemic neurotoxicity and reduce apoptosis by inhibiting the PERK-eIF2α-ATF4-CHOP axis [72]. Additionally, indole-3-propionic acid has been shown to reduce apoptosis by modulating the PERK-IRE1-ATF4-CHOP signaling pathway [73]. In regard to targeting the IRE pathway, it has been found that IRE1α siRNA inhibits ER stress, thereby reducing SCs’ apoptosis and alleviating DPN [74]. On the topic of targeting ER stress downstream signaling pathways, thioredoxin-1 was found to alleviate diabetic encephalopathy by reducing ER-stress-induced apoptosis through inhibiting apoptosis signal-regulating kinase 1 phosphorylation and decreasing JNK and caspase-12 expression. In addition, it can increase Nrf2 to inhibit NF-κB and reduce oxidative stress to alleviate misfold protein formation, thus reducing ER stress [75]. Furthermore, the traditional Chinese medicines Compound Qiying Granules and Tang-Luo-Ning were found to have similar effects [76,77].

Suppression of inflammation is a potential strategy for reducing cell apoptosis in DN. Curcumin, a well-documented anti-inflammatory agent, has been extensively researched for its therapeutic effects on diabetes and its complications [78]. In DN, studies have demonstrated that curcumin not only inhibits the NF-κB pathway to reduce inflammation and subsequently suppress SCs and spinal neuron apoptosis [79,80] but also enhances the expression of nerve growth factor [81], thereby protecting neurons and alleviating DN.

NMDA receptor antagonists present a potential approach for addressing apoptosis induced by glutamate-induced excitotoxicity. Animal model studies have shown that the use of NMDA receptor antagonists, such as memantine and neramexane, can alleviate diabetic neuropathic pain [82]. Meta-analyses of clinical studies also suggest that various NMDA receptor antagonists may have a role in relieving neuropathic pain in diabetic neuropathy [83]. However, there is currently a lack of detailed studies elucidating whether this alleviating effect is specifically due to the reduction in neuronal apoptosis. In addition, studies have shown that curcumin and allopregnanolone can reduce the levels of apoptosis-related molecules in DN. Although this effect may be related to their anti-inflammatory properties, the role of these substances in activating inhibitory neurotransmitter channels such as GABA, thereby counteracting glutamate-induced excitotoxicity, cannot be overlooked [42,84].

### 3.2. Autophagy

Autophagy is a physiological process in which cells utilize lysosomes to degrade and recycle their own cytoplasmic proteins and damaged organelles, thereby maintaining intracellular homeostasis [85]. In response to stress factors such as nutrient deprivation, the Unc-51-like autophagy-activating kinase 1 complex is activated, initiating autophagy. Under the regulation of the class III PI3K complex, which includes beclin-1, VPS34, VPS15, and Atg14L, this process promotes the recruitment of autophagy-related proteins, leading to the formation of a phagophore. Subsequently, with the involvement of two ubiquitin-like conjugation pathways, Atg12-Atg5-Atg16L and the microtubule-associated protein 1 light chain 3 system, the phagophore expands and engulfs targeted cellular components for degradation, leading to the formation of an autophagosome. Following the formation of the autophagosome, it fuses with a lysosome to form an autolysosome, where the engulfed materials are degraded. The breakdown products are then transported back to the cytoplasm for reuse, completing the cycle [86]. 

The effect of diabetes on the level of autophagy is controversial, with both overactivation and inhibition. A study found that the expression of autophagic biomarkers, Beclin and light chain 3, is decreased in diabetic mice, indicating a reduction in autophagy levels [87]. However, another study found that autophagy levels were upregulated in the DRG of diabetic rats [88]. Autophagy is involved in the regulation of various modes of cell death, and both high levels of autophagy and insufficient autophagy can induce cell death [89]. Therefore, modulating autophagy levels represents a potential strategy for inhibiting cell death in DN.

As mentioned previously, either excessive or low levels of autophagy may trigger cell death. Therefore, both promoting and inhibiting autophagy may reduce apoptosis. Regarding the promotion of autophagy, one study discovered that the TP53-inducible glycolysis and apoptosis regulator can reduce neuronal apoptosis and alleviate memory loss in the hippocampus by upregulating autophagy [90]. Another study found that Nesfatin-1 promotes autophagy to decrease apoptosis induced by high glucose in PC12 cells [91]. Furthermore, arctigenin can downregulate the AKT/mTOR pathway and activate autophagy to reduce apoptosis in the spinal cord neurons of STZ-induced diabetic mice [87]. In terms of inhibiting autophagy, one study found that salvianolic acid B can reduce SC apoptosis by downregulating autophagy through the inhibition of the JNK pathway [92]. Another study revealed that astragaloside IV mitigates myelin damage in DPN by upregulating microRNA-155 to inhibit the activation of the PI3K/Akt/mTOR pathway, thereby reducing SC apoptosis [93]. Future research on modulating autophagy to inhibit apoptosis should explore under what circumstances overactivation and impairment of autophagy occur, as well as the predisposition to these phenomena in different neural tissues and types of diabetes. Further studies could aid in more precisely determining whether to suppress or promote autophagy to reduce apoptosis.

### 3.3. Necrosis in DN 

#### 3.3.1. Pyroptosis 

Pyroptosis is a form of programmed cell necrosis associated with inflammatory responses. This type of cell death involves the activation of inflammasomes and gasdermin D (GSDMD), resulting in the formation of pores within the cell membrane. This leads to cell swelling, rupture, and the release of pro-inflammatory cellular contents [94]. Pyroptosis can be divided into the canonical pathway, mediated by caspase-1, and the non-canonical pathway, mediated by caspase-4/5 (caspase-11 in mice) [95]. As the effector of pyroptosis, GSDMD plays an important role in both pathways [96]. The canonical pathway is primarily activated by the detection of pathogen-associated molecular patterns or danger-associated molecular patterns by pattern recognition receptors. Upon activation, the inflammasome, a multi-protein complex, assembles. This complex typically comprises sensor proteins equipped with the pyrin domain, adaptor proteins featuring the caspase activation and recruitment domain such as apoptosis-associated speck-like protein, and effector protein caspase-1 [97]. Caspase-1 activated by inflammasomes cleaves GSDMD and processes pro-inflammatory cytokines pro-IL-1β and pro-IL-18 into their active forms. The N-terminal fragment of GSDMD forms pores in the cell membrane, leading to cell death and the release of IL-1β and IL-18 [98]. In the non-canonical pathway, lipopolysaccharide (LPS) directly interacts with caspase-4/5/11, leading to the cleavage of the GSDMD precursor form into its active fragment, N-GSDMD, and the subsequent formation of pores in the membrane. It is noteworthy that caspase-4/5/11 cannot cleave pro-IL-1β and pro-IL-18. However, the cleavage of GSDMD by these caspases leads to K^+^ efflux, which in turn induces the assembly of the NLRP3 inflammasome, resulting in pyroptosis [99]. Furthermore, the ATP released during cell pyroptosis can induce pyroptosis mediated by the purinergic ligand-gated ion channel seven (P2X7) receptor [100].

Diabetes can be considered a chronic inflammatory condition that leads to excessive pyroptosis. Increasing evidence supports the significant role of pyroptosis in the progression of diabetes and its complications. Studies on STZ-induced diabetic mice have shown a notable increase in the levels of pyroptosis-mediated proteins and pro-inflammatory cytokines IL-1β and IL-18 [101]. In DN, pyroptosis occurs in various cell types, leading to diverse symptoms. Research has demonstrated that diabetes induces pyroptosis in microglia and hippocampal neurons in the brain, resulting in brain injury and depression-like behaviors [102,103]. SCs can also undergo pyroptosis, leading to DPN, while similar processes in retinal microglia and enteric neurons lead to corresponding symptoms [104].

**Figure 3:**
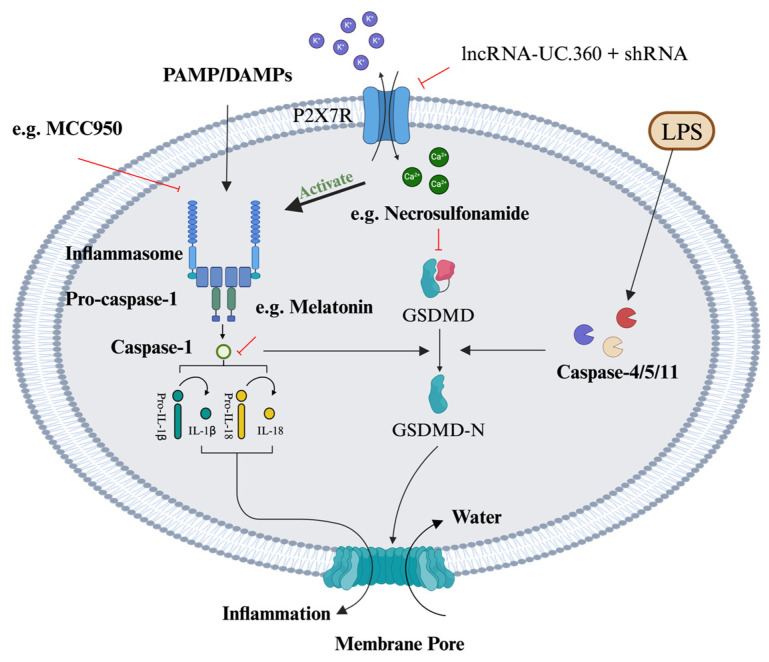
Mechanisms of pyroptosis and approaches for targeting pyroptosis. Activation of inflammasomes by PAMPs and DAMPs leads to the cleavage of pro-caspase-1 into active caspase-1. Caspase-1 then activates GSDMD, which forms membrane pores, resulting in cell swelling, rupture, and inflammation. Alternatively, LPS can activate caspase-4/5/11, which also cleaves GSDMD. Potassium efflux through P2X7 receptors and calcium influx are critical regulators of inflammasome activation. Therapeutic approaches include MCC950 (inflammasome inhibitor), necrosulfonamide (GSDMD inhibitor), melatonin (suppresses caspase-1), and lncRNA-UC.360+ shRNA (modulates upstream signaling). PAMPs, pathogen-associated molecular patterns; DAMPs, danger-associated molecular patterns; GSDMD, gasdermin D; P2X7R, purinergic ligand-gated ion channel seven receptor.

Recent studies have increasingly focused on the potential of targeting the pyroptosis pathway as a therapeutic strategy for DN. For targeting pyroptosis, experiences from other diseases can also be applied to DN. Currently, methods to inhibit cell pyroptosis primarily include inflammasome inhibitors, caspase inhibitors, GSDMD inhibitors, and antioxidants (Figure 3). MCC950, as a representative NLRP3 inflammasome inhibitor, has been discovered in previous research to alleviate ischemia–reperfusion injury in the brains of diabetic mice [105]. Furthermore, tauroursodeoxycholic acid can reduce pyroptosis in Schwann cells by inhibiting NLRP3 activation [106]. Another study found that upregulation of lipin2 in the hippocampus of diabetic encephalopathy mice alleviated cognitive dysfunction by inhibiting the JNK/ERK signaling pathway and reducing NLRP3 activation [107], which may also be a therapeutic target for pyroptosis. NLRP1 and NLRP4 have also been identified to play roles in neural damage and neuroinflammation in DN [102,108], suggesting that inhibitors targeting these two inflammasomes may also possess potential therapeutic effects. Regarding the inhibition of caspases, research has found that melatonin can reduce pyroptosis and autophagy in brain neurons of STZ-induced diabetic mice by targeting caspase-1 through miR-214-3p [101]. Although there are several recognized GSDMD inhibitors (e.g., Necrosulfonamide, LDC7559, Disulfiram), these all affect upstream signaling and lack sufficient specificity [109]. As for the effect of these GSDMD inhibitors in DN treatment, there is a lack of studies. In addition, it has also been found that lncRNA MSTRG.81401 can reduce pyroptosis in hippocampal neurons by inhibiting the P2X7 receptor/NLRP3/caspase1 pathway, suggesting that targeting the P2X7 receptor is also a way of reducing pyroptosis in DN [110]. For antioxidants, salvianolic acid B, loganin, and LncRNA-UC.360+ shRNA were found to reduce neuronal cell pyroptosis by downregulating intracellular ROS [111,112,113]. Notably, although inflammation is considered one of the factors leading to the formation of oxidative stress and pyroptosis, the effectiveness of commonly used non-steroidal anti-inflammatory drugs (NSAIDs) in treating DN is quite limited [114]. A study utilizing nano-delivery technology to improve the bioavailability of curcumin demonstrated that it could more effectively reduce cyclooxygenase-2 levels, thereby enhancing its neuroprotective effects [115]. NSAIDs primarily reduce inflammation by targeting cyclooxygenase. However, if NSAIDs cannot reach effective concentrations in neural tissue, they are unlikely to exert significant anti-inflammatory effects. Additionally, the mechanisms underlying DN are complex, and the single-target action of NSAIDs, coupled with their limited ability to scavenge reactive oxygen species (ROS), may contribute to their poor therapeutic efficacy.

#### 3.3.2. Necroptosis 

Necroptosis is a form of programmed necrotic cell death that involves the participation of receptor-interacting protein kinase 1 (RIPK1), RIPK3, and the mixed-lineage kinase domain-like protein (MLKL). When death receptor family ligands are upregulated, such as TNF receptors, this may trigger necroptosis. Upon receiving stimuli, RIPK1 recruits and phosphorylates RIPK3, which subsequently leads to the phosphorylation and translocation of MLKL to the plasma membrane, where it forms pores, resulting in membrane rupture and cell death [116]. Necroptosis plays a significant role in various diseases, including cancer, kidney diseases, and neurodegenerative disorders. Within the context of diabetes, there is a well-documented association between diabetic nephropathy and necroptosis [117]. However, the specific contribution of necroptosis to DN remains less understood. 

Recent studies have found that inhibiting the RIPK1/RIPK3/MLKL pathway can reduce necroptosis and alleviate DN. A recent study discovered that inhibition of RIPK3 by using a 5-hydroxytryptamine 4 receptor agonist can reduce necroptosis in the enteric neurons of diabetic mice [118]. Another study employed an S441A point mutation to modify the MLKL gene in mouse SCs, inhibiting the function of MLKL. It was found that in STZ-induced diabetic mice, myelin sheath decomposition was reduced, and necroptosis was inhibited. This provides a potential therapeutic target for treating DN by suppressing MLKL to decrease necroptosis.

### 3.4. Other Types of Cell Death

#### 3.4.1. Ferroptosis

Ferroptosis is a new type of regulated cell death characterized by iron-dependent lipid peroxidation, which causes the accumulation of lipid ROS, degradation of phospholipids containing polyunsaturated fatty acids (PUFAs) within the cell membrane, and ultimately cell death [119]. The process of ferroptosis primarily involves free iron overload, lipid peroxidation, and GSH depletion. Free iron catalyzes the generation of ROS through the Fenton reaction. ROS can attack PUFAs, leading to lipid peroxidation. Glutathione peroxidase 4 (GPX4) utilizes GSH as a cofactor to reduce lipid hydroperoxides, thus playing a protective role in preventing peroxidation. However, inactivation of the cystine/glutamate antiporter system Xc-, which leads to GPX4 inhibition and GSH depletion, shifts the balance toward cell death [120]. 

**Figure 4:**
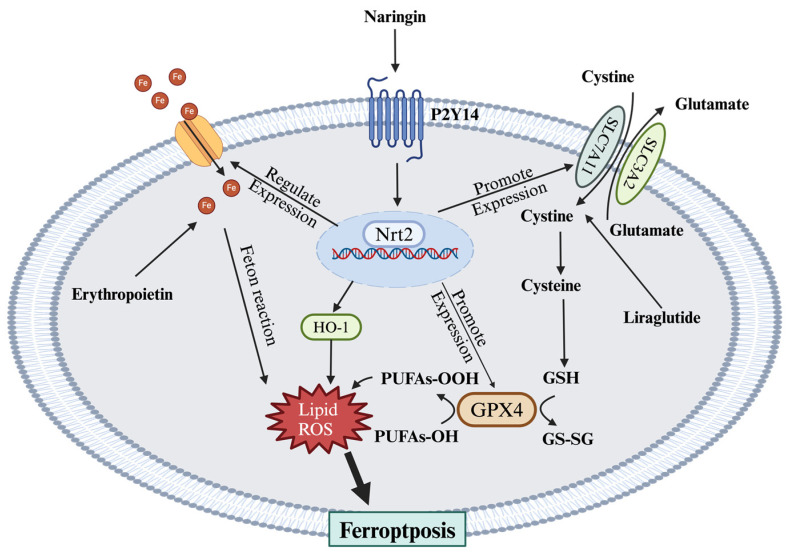
Mechanisms of ferroptosis and representative approaches for targeting ferroptosis. Ferroptosis is driven by lipid ROS and PUFA-OOH. Fpn transports Fe into the cell, where excess iron participates in the Fenton reaction, producing ROS. These ROS initiate and propagate the peroxidation of PUFAs in cell membranes, leading to the formation of PUFA-OOH. The accumulation of PUFA-OOH increases ROS levels and disrupts membrane integrity, resulting in ferroptosis. Therapeutic approaches aim to mitigate this process. Activation of the Nrf2 pathway enhances antioxidant defenses by upregulating HO-1 and GPX4. HO-1 modulates iron metabolism, reducing free iron levels, while GPX4 reduces PUFA-OOH to PUFA-OH. Erythropoietin decreases cellular iron levels. Liraglutide promotes the expression of the cystine/glutamate antiporter SLC7A11/SLC3A2, facilitating cystine uptake and GSH synthesis, which is crucial for GPX4 activity. Additionally, naringin activates Nrf2 via the P2Y14 receptor, further bolstering antioxidant defenses and inhibiting ferroptosis. PUFAs, polyunsaturated fatty acids; Fpn, ferroportin; Nrf2, nuclear factor erythroid 2-related factor 2; HO-1, heme oxygenase-1; GPX4, glutathione peroxidase 4; P2Y14, P2Y purinoceptor 14.

Ferroptosis in DN may be regulated by key proteins and pathways, such as the tumor suppressor protein p53, which promotes ferroptosis by downregulating SLC7A11 (solute carrier family 7 member 11) and SLC3A2 (solute carrier family 3 member 2), components of system X_c_^−^ [121]. In analyses of ferroptosis-related genes in diabetic feet, p53 has been found to be overexpressed in lesioned tissues [122]. In addition to p53, nuclear receptor coactivator 4, cysteinyl-tRNA synthetase, MAPK, and nicotinamide adenine dinucleotide phosphate oxidase have been identified to positively regulate ferroptosis by promoting iron accumulation, lipid peroxidation, and ROS production. Conversely, Nrf2 can negatively regulate ferroptosis by enhancing cellular antioxidant defenses and maintaining iron homeostasis, thereby protecting cells against ferroptosis [123]. Nrf2 can activate the transcription of genes associated with the antioxidant response element, which regulate ferroptosis by encoding a variety of proteins and enzymes crucial for reducing oxidative stress and inhibiting ferroptosis. Nrf2 modulates iron metabolism and resists iron overload to suppress ferroptosis. This process can be facilitated by increasing the expression of ferroportin (Fpn), which promotes iron transport out of the cell, and by enhancing the expression of ferrochelatase, which promotes the binding of iron ions to protoporphyrin. Additionally, Nrf2 promotes the transcription of several target genes, including GPX4, HO-1, and SLC7A11, thereby enhancing the cellular antioxidant defenses and inhibiting ferroptosis (Figure 4). These complex regulatory mechanisms highlight potential targets for therapeutic intervention in diseases in which ferroptosis plays a pathogenic role.

Recent studies have highlighted the critical role of the Nrf2 signaling pathway in modulating cell ferroptosis in DN. It has been found that hyperglycemia can trigger ferroptosis in SC by suppressing the Nrf2 signaling pathway [124]. Therefore, targeting Nrf2 to reduce ferroptosis and thereby alleviate DN is a potential therapeutic modality, and several studies have shown positive results. A study has shown that the traditional Chinese medicine naringin can alleviate diabetic cardiac autonomic neuropathy by targeting the P2Y_14_ receptor to reduce the expression of inflammatory factors and promote the antioxidant Nrf 2/Gpx4 pathway [125]. Another study found that CircRNA-itchy E3 ubiquitin protein ligase activates the Nrf2 pathway to reduce ferroptosis by recruiting TATA box-binding protein associated factor 15 [126]. In addition, activation of the Nrf2 pathway to reduce ferroptosis plays a role in diabetes-associated cognitive dysfunction, and one study demonstrated that artemisinin activates the Nrf2 signaling pathway, thereby upregulating HO-1, GPX4, and GSH, which mitigates ferroptosis in the hippocampal neurons of mice, resulting in symptomatic improvement [127]. Dendrobine has also been found to inhibit neuronal ferroptosis in the cerebral cortex and hippocampal regions of mice by activating the Nrf2/GPX4 pathway [128]. Sinomenine can upregulate the EGF/Nrf2/HO-1 pathway through the intestinal microbiota–gut–brain axis to inhibit hippocampal neurons’ ferroptosis [129]. Research has found that activation of AMPK can inhibit neuronal ferroptosis [130]. Another study on caveolin-1 indicates that this effect is achieved through the phosphorylation of AMPK, activating Nrf2, which then upregulates Fpn to regulate iron metabolism and inhibit ferroptosis. Overexpression of caveolin-1 can enhance this process [131]. In addition to the effects mediated by the Nrf2-related pathway, some studies have also demonstrated efficacy by targeting processes related to ferroptosis. Erythropoietin can reduce iron overload and lipid peroxidation, thereby inhibiting ferroptosis in hippocampal neurons [132]. Liraglutide promotes the expression of GPX4 and SLC7A11 to inhibit ferroptosis [133]. Downregulating the Fpn encoding gene SLC40A1 can also inhibit ferroptosis involved in cognitive impairment in DN [134]. The peroxisome proliferator-activated receptor-alpha agonist gemfibrozil can inhibit ferroptosis in astrocytes and alleviate cognitive impairment by restoring the canonical xCT/GPX4-regulated ferroptosis pathway and preventing iron overload [135]. Additionally, inhibiting the JNK-inflammatory factor pathway can also mitigate ferroptosis in hippocampal neurons [136]. This indicates that inflammatory responses play a significant role in ferroptosis within DN, potentially serving as a viable therapeutic target.

#### 3.4.2. Parthanatos 

Parthanatos is a form of programmed cell death mediated by PARP-1, triggered by DNA damage. Upon DNA damage, PARP-1 becomes hyperactivated, leading to the excessive production of PAR polymers. The accumulation of these polymers depletes cellular NAD+ and ATP, resulting in cellular energy failure. Crucially, an excess of PAR prompts the release of apoptosis-inducing factor (AIF) from the mitochondria into the nucleus, causing the cleavage of genomic DNA into large fragments and ultimately leading to cell death [137]. In DN, current experimental evidence in vitro suggests that a hydrogen-rich medium can decrease PAR levels and prevent the translocation of AIF in rat SCs, thereby indicating its potential to suppress parthanatos [138].

## 4. Future Perspectives

There are various modes of cell death, and in DN, certain cells, such as SCs, may exhibit multiple forms of cell death simultaneously, including apoptosis, pyroptosis, ferroptosis, and necroptosis. Future research is necessary to determine which mode of cell death predominates in specific neural tissues, as this knowledge could enhance the selection of targeted therapeutic interventions. Moreover, therapeutic approaches to cell death, with some substances like melanin acting both as antioxidants and modulators of autophagy [101,139,140], suggest that future studies should investigate whether combining drugs that inhibit cell death through different mechanisms could yield improved outcomes. In addition, cell death mechanisms are a hot research topic, and some new cell death mechanisms have been identified. For example, cuproptosis may play a role in diabetes and its complications [141], and whether other cell death mechanisms exist in DN remains to be ascertained.

## 5. Conclusions

In summary, cell death is a significant contributing factor to DN. Oxidative stress, mitochondrial dysfunction, inflammation, ER stress, and autophagy in DN can lead to various forms of cell death, including apoptosis, pyroptosis, and ferroptosis. Targeting the initiating factors of different cell death modes may represent a potential therapeutic strategy (Table 1). However, the mechanisms underlying DN are complex, and multiple forms of cell death may be involved concurrently. Therefore, identifying the dominant mode of cell death and developing more targeted pharmaceutical interventions are essential.

## Figures and Tables

**Table 1:** Mechanism of different death programs and the involved molecules.

Mechanism of Cell Death	Key Players	Involvement in DN	Potential Therapeutic Targets	Agents	References
Apoptosis	BCL-2 family proteins, Cytochrome c, Caspases	Apoptosis occurs in neurons and glial cells, leading to demyelination and neuronal damage	Antioxidants, Inhibition of death pathways, Anti-inflammatory agents	Alpha-lipoic acid (ALA)	[59,60,61]
Autophagy	Autophagy-related proteins (ATGs), Beclin-1, LC3	Controversial effect in DN, both overactivation and inhibition reported	Modulation of autophagy levels, Inhibition of mTOR pathway	Arctigenin (inhibitor of mTOR)	[87]
Pyroptosis	Inflammasomes, Caspase-1, GSDMD	Associated with neuroinflammation and neuronal damage in DN	Inflammasome inhibitors, Caspase inhibitors, GSDMD inhibitors	MCC950 (NLRP3 inflammasome inhibitor)	[105]
Ferroptosis	GPX4, System Xc-, Iron-metabolism-related proteins	Potential role in neuronal damage in DN	Activation of Nrf2 pathway, Inhibition of lipid peroxidation	Liraglutide (promotes GPX4 and SLC7A11 expression)	[133]
Necroptosis	RIPK1, RIPK3, MLKL	Less understood in DN, but associated with diabetic nephropathy	Inhibition of RIPK1/RIPK3/MLKL pathway	N/A	N/A
Parthanatos	PARP-1, AIF	Triggered by DNA damage in DN	Reduction in PAR levels, Prevention of AIF translocation	Hydrogen-rich medium	[138]

N/A: Not Applicable.

## Data Availability

Not applicable.
